# TIMP-2 Interaction with MT1-MMP Activates the AKT Pathway and Protects Tumor Cells from Apoptosis

**DOI:** 10.1371/journal.pone.0136797

**Published:** 2015-09-02

**Authors:** Cristina Valacca, Evelyne Tassone, Paolo Mignatti

**Affiliations:** 1 Department of Cardiothoracic Surgery, New York University School of Medicine, New York, New York, United States of America; 2 Department of Medicine, New York University School of Medicine, New York, New York, United States of America; 3 Department of Cell Biology, New York University School of Medicine, New York University School of Medicine, New York, New York, United States of America; Stony Brook University, UNITED STATES

## Abstract

Membrane-type 1 matrix metalloproteinase (MT1-MMP), a transmembrane proteinase with an extracellular catalytic domain and a short cytoplasmic tail, degrades a variety of extracellular matrix (ECM) components. In addition, MT1-MMP activates intracellular signaling through proteolysis-dependent and independent mechanisms. We have previously shown that binding of tissue inhibitor of metalloproteinases-2 (TIMP-2) to MT1-MMP controls cell proliferation and migration, as well as tumor growth *in vivo* by activating the Ras—extracellular signal regulated kinase-1 and -2 (ERK1/2) pathway through a mechanism that requires the cytoplasmic but not the proteolytic domain of MT1-MMP. Here we show that in MT1-MMP expressing cells TIMP-2 also induces rapid and sustained activation of AKT in a dose- and time-dependent manner and by a mechanism independent of the proteolytic activity of MT1-MMP. Fibroblast growth factor receptor-1 mediates TIMP-2 induction of ERK1/2 but not of AKT activation; however, Ras activation is necessary to transduce the TIMP-2-activated signal to both the ERK1/2 and AKT pathways. ERK1/2 and AKT activation by TIMP-2 binding to MT1-MMP protects tumor cells from apoptosis induced by serum starvation. Conversely, TIMP-2 upregulates apoptosis induced by three-dimensional type I collagen in epithelial cancer cells. Thus, TIMP-2 interaction with MT1-MMP provides tumor cells with either pro- or anti-apoptotic signaling depending on the extracellular environment and apoptotic stimulus.

## Introduction

Membrane-type 1 matrix metalloproteinase (MT1-MMP, MMP-14), a transmembrane proteinase with an extracellular catalytic site and a 20-amino acid cytoplasmic domain, degrades a variety of extracellular matrix (ECM) components and activates the proenzyme forms of MMP-2 and MMP-13 [1]. Based on these features MT1-MMP has been implicated as a central component of the proteolytic mechanisms of a variety of physiological and pathological processes, including tumor invasion, metastasis and angiogenesis [2,3]. However, increasing evidence now shows that, in addition to remodeling the ECM, MT1-MMP is a multifunctional protein that controls intracellular signaling by proteolysis-dependent and independent mechanisms. MT1-MMP controls a variety of signaling pathways and cell functions, including necrosis/apoptosis [4], NF-κB-mediated cyclooxygenase-2 (COX-2) expression [5,6], hypoxia-inducible factor-1 alpha (HIF-1α)-mediated response of tumor cells to hypoxia [7], and vascular smooth muscle cell differentiation [8,9]. MT1-MMP controls fibroblast growth factor-2 (FGF-2) signaling by several mechanisms in diverse cell types. It forms a complex with FGF receptor (FGFR)-4 [10], and potentiates FGF-2 induction of corneal angiogenesis by modulating FGF-2 activation of intracellular signaling [11]. In calvarial osteoblasts MT1-MMP upregulates FGF signaling by shedding ADAM-9, which in turn cleaves FGFR-2 [12]. However, in tumor cells MT1-MMP downregulates FGF signaling by reducing FGF binding to the cell surface [13]. In skeletal stem cells MT1-MMP controls cell lineage commitment through β1-integrin/Rho GTPase-mediated activation of the YAP and TAZ transcriptional coactivators [14].

The proteolytic activity of MT1-MMP is physiologically inhibited by tissue inhibitor of metalloproteinase-2 (TIMP-2), a member of a multigene family of proteins (TIMP-1 through -4) that bind non-covalently to the catalytic domain of MMPs in a 1:1 molar ratio and specifically inhibit their activity [15]. TIMP-2 also controls several cell functions including migration, proliferation and apoptosis through MMP-dependent and -independent mechanisms [16–20]. It inhibits FGF-2-induced endothelial cell proliferation [21], suppresses the mitogenic activity of epidermal growth factor (EGF) [22] and inhibits angiogenic factor-induced endothelial cell proliferation and angiogenesis by increasing a protein tyrosine phosphatase activity associated with FGF and VEGF receptors [23]. Thus, TIMP-2 is a bifunctional protein with both growth factor and anti-proteolytic activities.

TIMP-2 and MT1-MMP are often co-expressed in normal or pathological tissues. Experimental and clinical data have implicated MT1-MMP and TIMP-2 in tumor progression. MT1-MMP acts as an oncogene, stimulates tumor cell invasion and metastasis [3,24,25], and high levels of MT1-MMP are associated with a variety of human aggressive malignancies [26]. In human breast carcinoma MT1-MMP levels correlate significantly with lymph node and distant metastasis, clinical stage and tumor size [27]. Paradoxically, in a variety of tumors high levels of TIMP-2—which inhibits several MMPs including MT1-MMP—also correlate with a poor prognosis. Indeed, a negative outcome of certain malignancies correlates more closely with TIMP-2 levels than with MT1-MMP levels [28–35], and high TIMP-2 levels in primary breast carcinomas are associated with the development of distant metastases [30,36].

We have shown that TIMP-2 binding to MT1-MMP rapidly activates extracellular signal-regulated kinase-1 and -2 (ERK1/2) by a non-proteolytic mechanism that upregulates cell proliferation and migration, as well as tumor growth [37]. This effect is mediated by the cytoplasmic domain and independent of the proteolytic activity of MT1-MMP. Here we report that in MT1-MMP expressing human tumor cells TIMP-2 also induces rapid and sustained activation of AKT in a dose- and time-dependent manner, and by a mechanism independent of the proteolytic activity of MT1-MMP. The signaling activated by TIMP-2 binding to MT1-MMP protects the cells from apoptosis induced by serum starvation through both the ERK1/2 and AKT pathways. Conversely, TIMP-2 – MT1-MMP interaction upregulates apoptosis induced by three-dimensional type I collagen. Thus, TIMP-2 interaction with MT1-MMP provides tumor cells with either pro- or anti-apoptotic signaling depending on the extracellular environment and apoptotic stimulus.

## Materials and Methods

### Cells and Culture Media

Human MCF-7 breast adenocarcinoma cells and MCF-7 cells stably transfected with MT1-MMP cDNA under control by the tetracycline resistance transactivator in the Tet-Off conformation have been described [37]. Human MDA-MB-435 melanoma cells were from American Type Culture Collection (ATCC; HTB-129). MCF-7 and MDA-MB-435 cells were grown in DMEM supplemented with 10% fetal calf serum (FCS), 2 mM L-glutamine, 100 U/ml penicillin, and 100 μg/ml streptomycin.

### Cell Treatments

Subconfluent cells grown for 24 h in medium containing 0.5% FCS with or without DOX (1 μg/ml) were incubated with the indicated concentrations of recombinant human TIMP-2 (Peprotech, Rocky Hill, NJ, USA) for 15 min. TIMP-2 was added to the cultures in a volume of 1 μl without changing the medium. An equivalent volume of medium without TIMP-2 was added as a control. Where indicated, Ilomastat (GM6001, 50 μM; Millipore, Billerica, MA, USA), PD173074 (5 nM or 21.5 nM; Sigma-Aldrich, St. Louis, MO, USA), U0126 (10 μM; Promega, Madison, WI, USA) or LY294002 (10 μM; BioVision, Milpitas, CA, USA) were added to the medium 15 min (Ilomastat and PD173074) or 30 min (U0126 and LY294002) before TIMP-2 addition.

### Apoptosis Induction

MCF-7 cell transfectants were grown in medium containing 10% FCS; after 24 h (day 0) the medium was replaced with serum-free medium supplemented with TIMP-2 with or without the indicated additions. TIMP-2, U0126 or LY294002 were added every other day without changing the medium. The cells were collected at day 7 for Western blotting analysis of PARP degradation. 3D type I collagen gels (2 mg/ml) were made from a neutralized solution of bovine type I collagen (BD Biosciences, San Jose, CA, USA). Cells suspended in medium containing 10% FCS were added to the collagen mix prior to gelling, and gels were cast in 12-well plates. TIMP-2 was added every other day. The cells were recovered from 3D cultures at day 7 by dissolving the gels in 2 mg/ml bacterial collagenase (Sigma-Aldrich). The reaction was stopped with FCS, and the cells were immediately used for further analysis. MDA-MB-435 cells grown on glass coverslips were transiently transfected with MT1-MMP siRNA (as described below) 24 h and 48 h after seeding. Eight hours after the second transfection the culture medium was changed to serum-free medium with or without addition of TIMP-2 (100 ng/ml). TIMP-2 or an equivalent volume of control medium was subsequently added every 24 h in the following 2 days. The cells were then fixed and stained for characterization of apoptotic nuclei as described below.

### Chromatin Morphology Assay

MDA-MB-435 cells on glass coverslips were serum-starved to induce apoptosis as described above. Apoptosis was characterized by staining the nuclei with Hoechst 33342 (Sigma; 10 mg/ml) to detect the condensed or fragmented chromatin pattern characteristic of apoptotic cells. For each experiment, 300 to 400 nuclei from 5 random fields of each coverslip were examined at high magnification (400X) with a Zeiss Axioskope 2 photomicroscope. The results are expressed as mean number of apoptotic nuclei ± sd / 10 X field, determined in three separate experiments.

### Transient Transfection

The plasmids encoding wt and mutant MT1-MMP have been described [37]. The dominant negative mutant of Ras (HRASN17) was a kind gift of Dr. Mark R. Philips (NYU School of Medicine, New York, USA). The constructs (4 μg) were transiently transfected into sub-confluent cells in 6-well plates using 10 μl of Lipofectamine (Invitrogen, Grand Island, NY, USA) according to the manufacturer’s instructions. Twenty-four hours after transfection, the cells were incubated with medium containing 0.5% FCS for additional 24 h, and immediately used for the experiments. MT1-MMP siRNA (siGENOME SMART) and control siRNA pools (Dharmacon GE Life Sciences, Piscataway, NJ) were transiently transfected into subconfluent MDA-MB-435 cells in 6-well plates using 5 μl of Lipofectamine according to the manufacturer’s instructions. For the AKT activation experiments, twenty-four hours after transfection the cells were incubated with medium containing 0.5% FBS for additional 24 h before being treated with TIMP-2. For the apoptosis experiments the cells were treated as described above (Apoptosis induction).

### Western Blotting and Antibodies

Cells were lysed in RIPA buffer (150 mM NaCl, 1% Igepal, 0.5% sodium deoxycholate, 0.1% SDS in 50 mM Tris-HCl, pH 8.0) containing protease (Complete, Roche, Indianapolis, IN, USA) and phosphatase inhibitors (PhosSTOP, Roche). The lysates were sonicated and centrifuged (14,000 rpm for 15 min at 4°C). Cell extract protein (40 μg) was electrophoresed in SDS/10% or 12% polyacrylamide gels, and analyzed by Western blotting. The following antibodies and predetermined dilutions were used: MT1-MMP (Millipore), p-ERK1/2, p-AKT and cleaved PARP (Cell Signaling Technology; Danver, MA; USA) 1:1,000; ERK1/2 (Cell Signaling Technology) 1:20,000; AKT (Cell Signaling Technology) 1:10,000; β-tubulin (Sigma-Aldrich) 1:100,000; horseradish peroxidase-conjugated anti-mouse and anti-rabbit (Jackson ImmunoResearch Laboratories, West Grove, PA, USA) 1: 2,500–20,000. In most experiments membranes probed with a set of first and second antibody were stripped of the antibodies with a mild stripping buffer (20 mM Glycine, 0.1% SDS, 1% Tween 20, pH 2.2) for 30 min at room temperature with gentle agitation, and reprobed with a second set of antibodies to different antigen.

### Densitometry

Quantitative analysis of Western blot bands was performed with ImageJ 10.2 software (National Institutes of Health). Data are shown as mean ± SE of the ratio between the reading of the sample and that of the corresponding loading control.

### Statistical Analysis

The data were analyzed using the Students' *t* test. A p value ≤ 0.05 was considered as significant.

## Results

### TIMP-2 Interaction with MT1-MMP Induces AKT Activation

**Fig 1:**
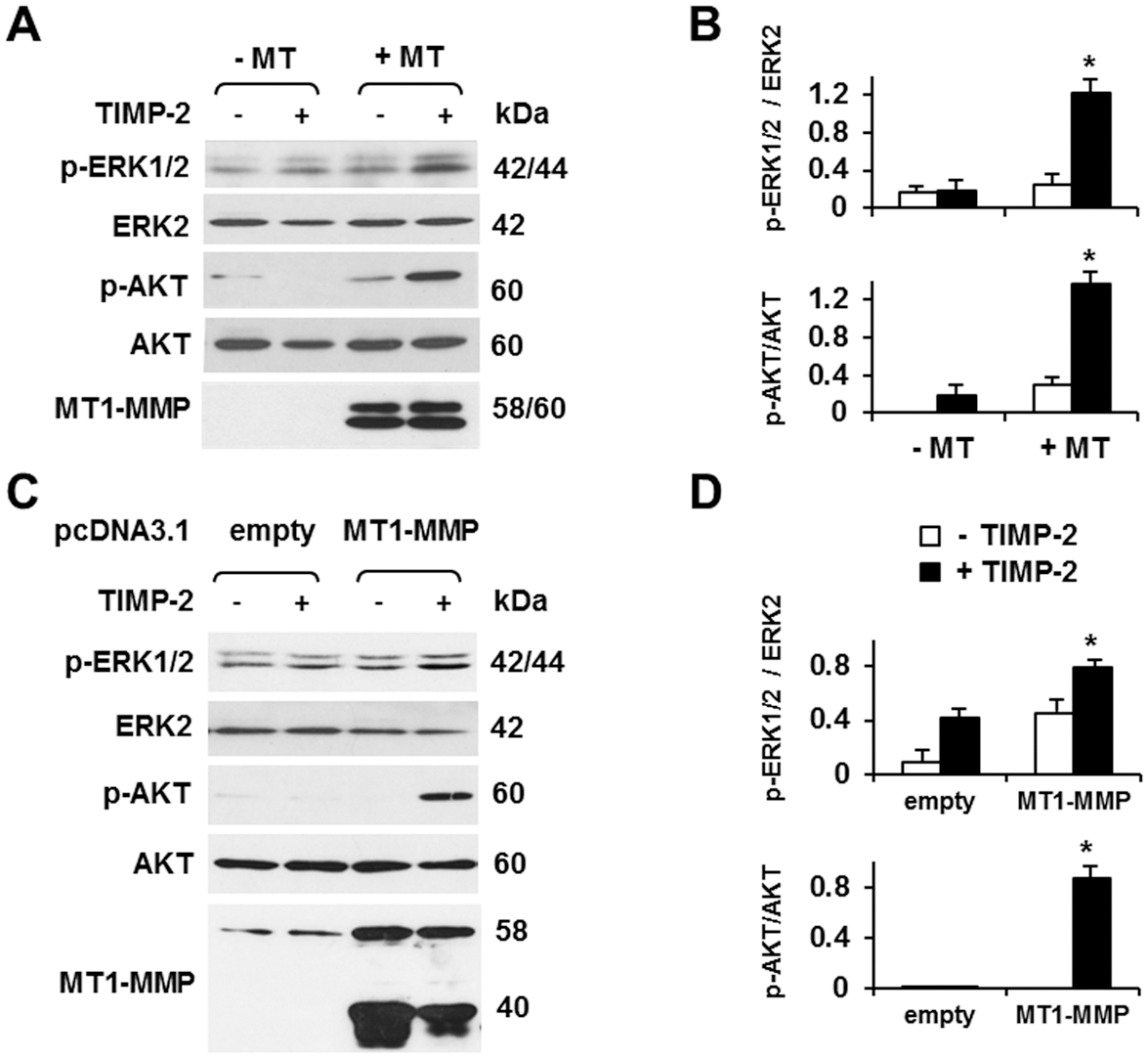
TIMP-2 induces ERK1/2 and AKT activation in MT1-MMP expressing MCF-7 cells. Western blotting analysis of ERK1/2 and AKT activation (p-ERK1/2; p-AKT) and MT1-MMP expression. **A**. MT1-MMP Tet-Off MCF-7 cells grown for 24 h in the presence (-MT) or absence of DOX (1 μg/ml; +MT) in medium containing 0.5% FCS were treated with TIMP-2 (100 ng/ml) for 15 min. Total ERK2 and AKT are shown as loading controls. The MT1-MMP panel shows the 60 kDa proenzyme and the 58 kDa active proteinase forms. **B**. Densitometric analysis of the p-ERK1/2 and p-AKT bands shown in A, normalized to the corresponding ERK2 and AKT controls, respectively. **C**. MCF-7 cells were transiently transfected with MT1-MMP cDNA or control empty vector. After 24 h starvation in 0.5% FCS medium the cells were treated with TIMP-2 (100 ng/ml) for 15 min. The MT1-MMP panel shows the 58 kDa active proteinase and ~ 40 kDa degradation products derived from autocatalysis. Total ERK2 and AKT are shown as loading controls. **D**. Densitometric analysis of the p-ERK1/2 and p-AKT bands shown in C, normalized to the corresponding ERK2 and AKT controls, respectively. *, *p* ≤ 0.05; + TIMP-2 *vs*. the corresponding – TIMP-2 sample. The experiment shown in panel A was repeated multiple times with comparable results. The experiment shown in panel C was repeated twice with similar results.

We have previously shown that TIMP-2 binding to MT1-MMP activates the Ras-ERK1/2 pathway by a proteolysis-independent mechanism [37]. To study the potential role of MT1-MMP and TIMP-2 in AKT activation we used human MCF-7 mammary carcinoma cells stably transfected with MT1-MMP under control by the tetracycline resistance transactivator (Tet-Off) [37]. In the presence of doxycycline (DOX; 1 μg/ml) in the culture medium these cells express virtually undetectable levels of MT1-MMP, like the parental non-transfected cells; removal of DOX induces expression of high levels of MT1-MMP [37,38] (Fig 1A and 1B). Our previous work had shown that ERK1/2 activation in MT1-MMP expressing cells becomes detectable after 5 min and peaks at 15 min of TIMP-2 (100 ng/ml) treatment [37]. Therefore, we incubated our MT1-MMP Tet-Off transfectants with TIMP-2 (100 ng/ml) for 15 min, and characterized AKT activation by Western blotting with antibody to phosphorylated AKT. TIMP-2 treatment of cells devoid of MT1-MMP (grown in the presence of DOX), or MT1-MMP expression in the absence of TIMP-2 resulted in modest AKT activation. However, TIMP-2 treatment of MT1-MMP expressing cells strongly upregulated AKT activation. Similarly, consistent with our previous findings [37], TIMP-2 induced ERK1/2 activation in MT1-MMP expressing cells but had no such effect on cells devoid of MT1-MMP (Fig 1A and 1B). These findings indicated that TIMP-2 interaction with MT1-MMP activates AKT as well as ERK1/2.

To confirm these results we transiently transfected MCF-7 cells with MT1-MMP cDNA or with the empty vector as a negative control. Consistent with our previous results, addition of TIMP-2 to the culture medium strongly induced AKT activation in the MT1-MMP transfectants but had no such effects on the control cells (Fig 1C and 1D).

**Fig 2:**
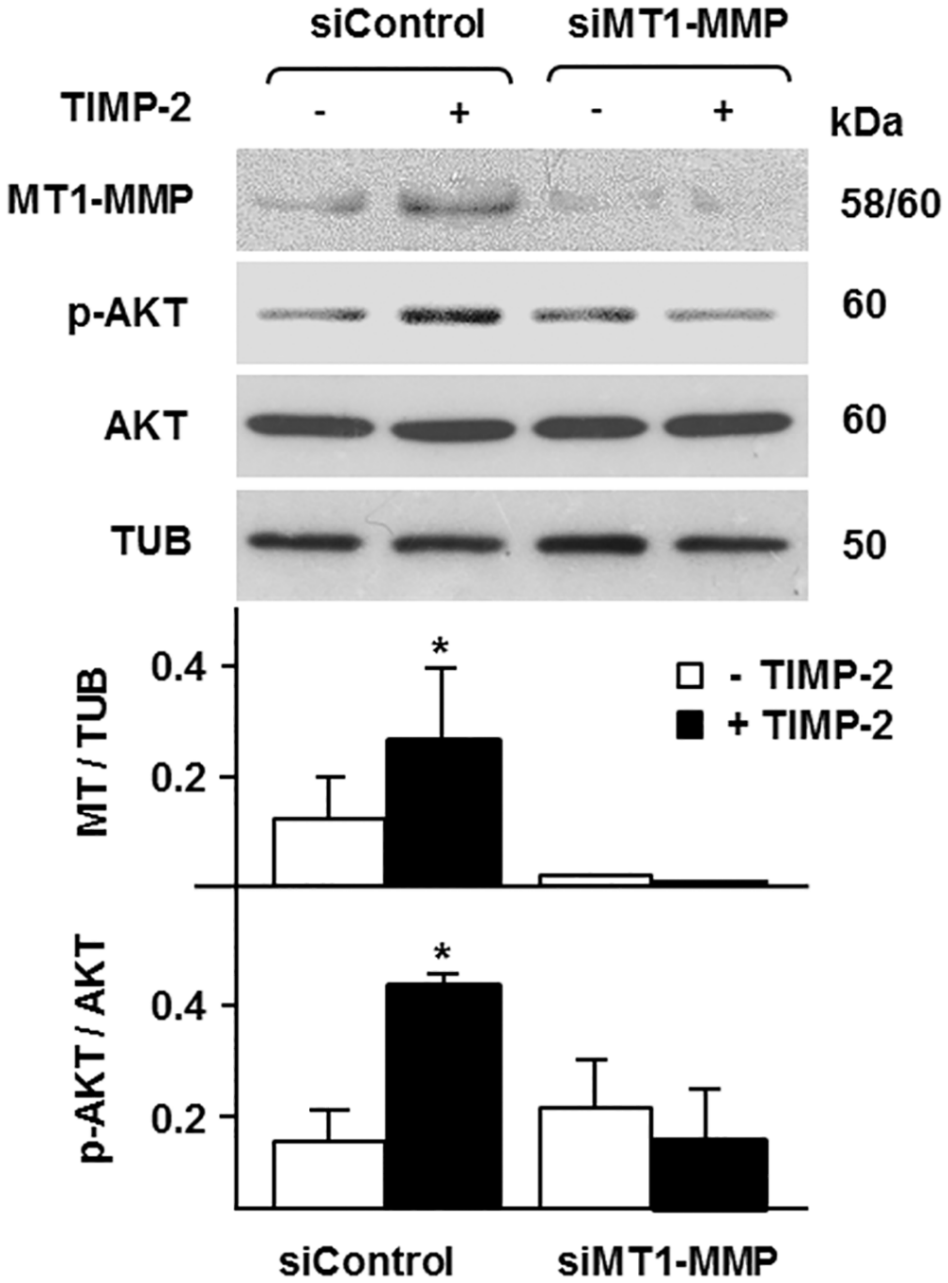
Downregulation of MT1-MMP blocks TIMP-2 activation of AKT in MT1-MMP expressing MDA-MB-435 cells. Western blotting analysis of AKT activation (p-AKT) and MT1-MMP expression in MDA-MB-435 cells transiently transfected with MT1-MMP siRNA (siMT1-MMP) or control, scrambled siRNA (siControl), and incubated with TIMP-2 (100 ng/ml) for 15 min. Total AKT and β-tubulin (TUB) are shown as loading controls. The lower panel shows the densitometric analysis of the MT1-MMP and p-AKT bands normalized to the corresponding TUB and AKT controls, respectively. *, *p* ≤ 0.05; + TIMP-2 *vs*. the corresponding – TIMP-2 sample. This experiment was repeated twice with comparable results.

To investigate whether TIMP-2 activation of AKT is a unique feature of MCF-7 cells or results from the high levels of MT1-MMP expressed by the transfected cells, we characterized the effect of TIMP-2 on AKT activation in human MDA-MB-435 melanoma cells, which constitutively express MT1-MMP. To analyze the requirement for MT1-MMP we transfected these cells with MT1-MMP siRNA or control, scrambled siRNA. Forty-eight hours later the cells were treated with TIMP-2, and analyzed for MT1-MMP expression and AKT activation. MT1-MMP expression was reduced by approximately 80% in the cells transfected with MT1-MMP siRNA relative to the control siRNA transfectants (Fig 2). TIMP-2 addition to the culture medium of control siRNA transfectants resulted in increased level of MT1-MMP, an effect that reflects the stabilization and accumulation of the relatively low amount of active MT1-MMP on the cell surface [39]. Consistent with our previous results, addition of TIMP-2 to the culture medium of control siRNA-transfected cells induced rapid (15 min) activation of AKT. Conversely, TIMP-2 had no such effect in MT1-MMP siRNA transfectants, which expressed almost undetectable levels of MT1-MMP (Fig 2).

**Fig 3:**
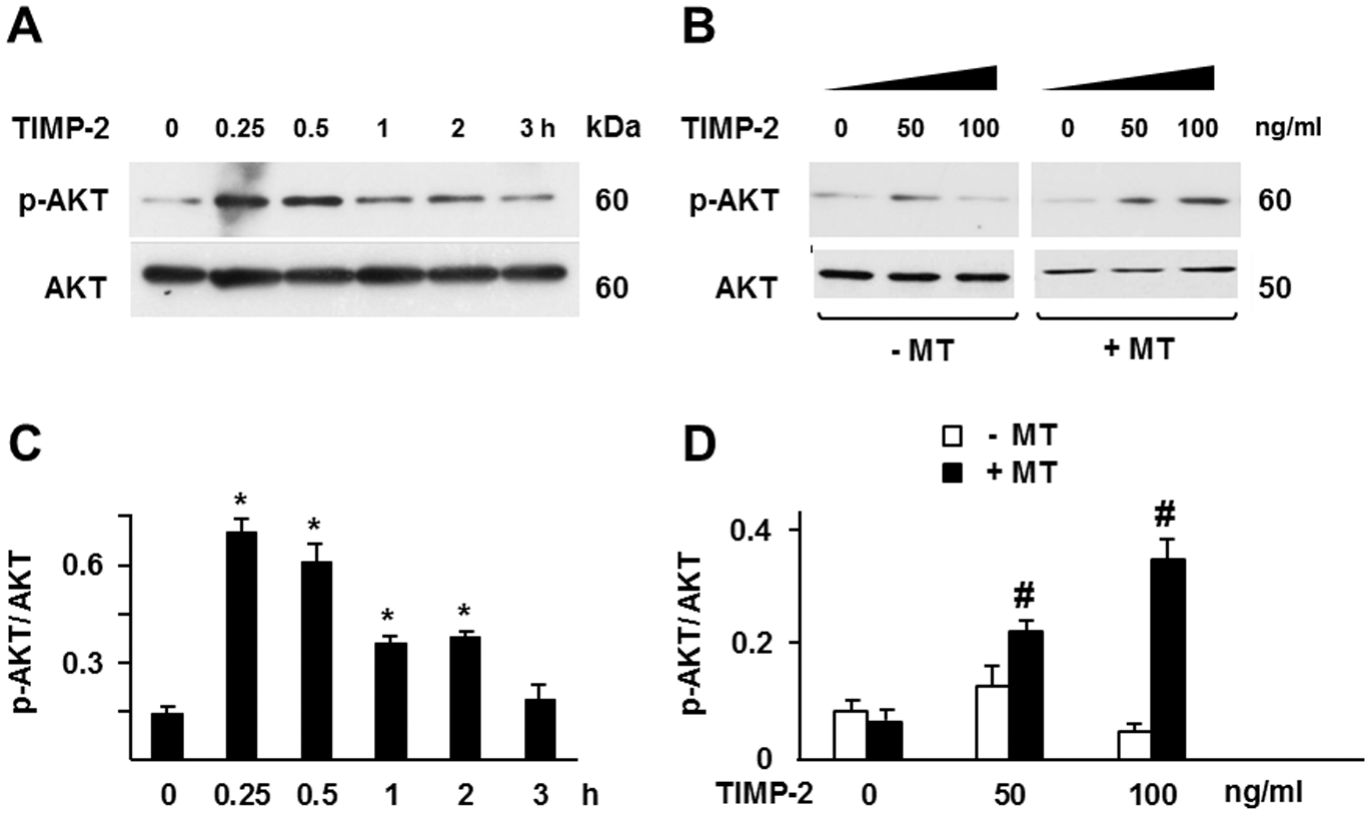
TIMP-2 induces AKT activation in a dose- and time-dependent manner. Western blotting analysis of AKT activation (pAKT) in MT1-MMP Tet-Off MCF-7 cells incubated with (**A**) TIMP-2 (100 ng/ml) for the indicated times or (**B**) with the indicated concentrations of TIMP-2 for 15 min. The cells shown in panel A were grown in the absence of DOX; the cells shown in panel B were grown either in the presence (-MT) or in the absence (+MT) of DOX. Total AKT is shown as a loading control. **C** and **D**. Densitometric analysis. *, *p* ≤ 0.05, *vs*. time 0; #, *p* ≤ 0.05, + MT1-MMP *vs*. the corresponding – MT1-MMP sample. These experiments were repeated twice with similar results.

AKT activation in MT1-MMP expressing cells occurred within 15 min of TIMP-2 addition and high levels of active AKT persisted for at least 2 h (Fig 3), showing that TIMP-2 interaction with MT1-MMP results in rapid and sustained activation of AKT. Importantly, AKT activation was induced by TIMP-2 concentrations of 50 ng/ml or 100 ng/ml (2.4–4.8 nM). These concentrations are similar to those found in tissues or biological fluids (0.5–5.0 nM) [40–43] and to the K_D_ of TIMP-2 for MT1-MMP (0.77–2.54 nM) [44,45], indicating that the effect of TIMP-2 on AKT activation can occur under physiological conditions.

### TIMP-2 Activation of AKT Is Independent of the Proteolytic Activity of MT1-MMP

**Fig 4:**
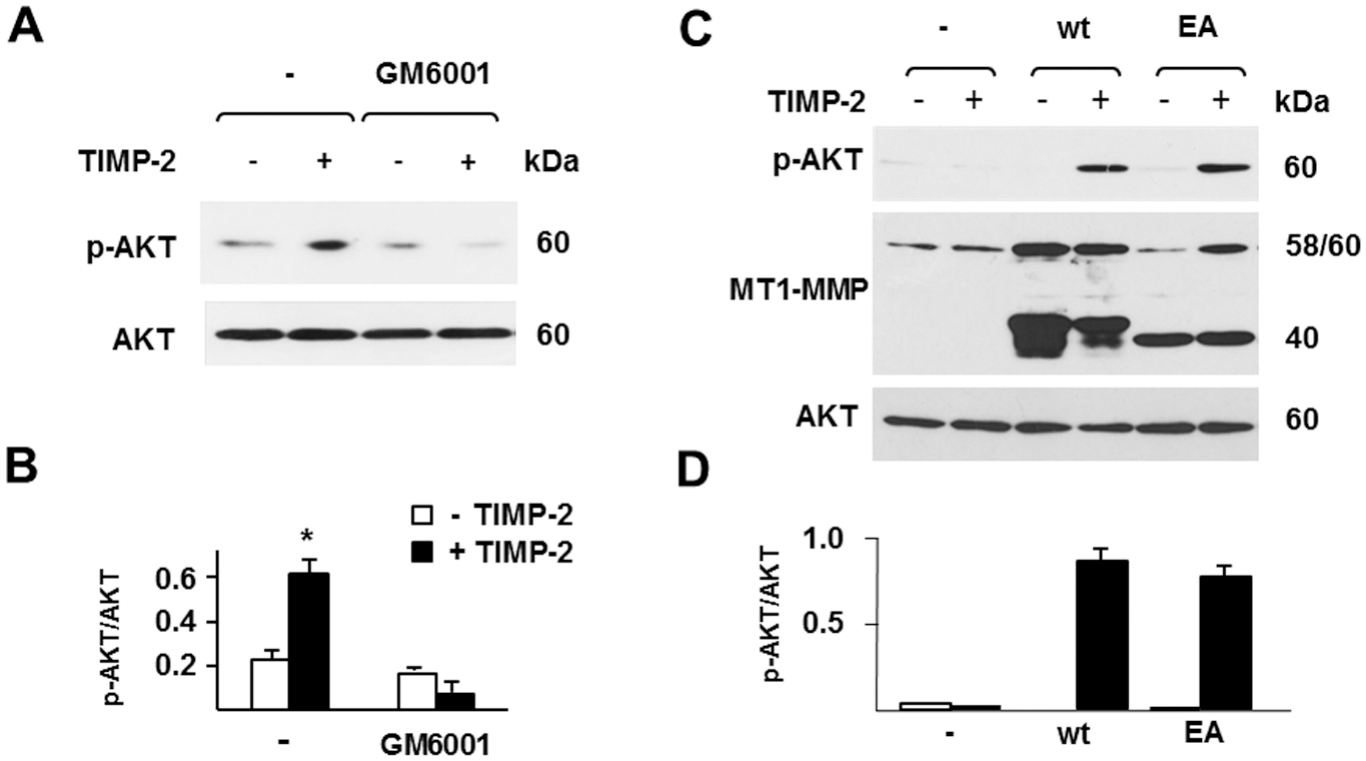
TIMP-2 induction of AKT activation does not require the proteolytic activity of MT1-MMP. **A and C**. Western blotting analysis of AKT activation (pAKT) in (**A**) MT1-MMP-expressing Tet-Off MCF-7 cells pretreated with Ilomastat (GM6001; 50 μM) or control vehicle (-) for 15 min, and (**C**) MCF-7 cells transiently transfected with the cDNAs for either wt MT1-MMP (wt) or MT1-MMP E240A (EA), and treated with TIMP-2 (100 ng/ml) or control medium (-) for 15 min. Cell protein extracts of the transient transfectants (C) were also analyzed for MT1-MMP expression. In the MT1-MMP panel the 60 kDa and the 58 kDa bands represent the proenzyme and the active proteinase forms, respectively; the 40 kDa M_r_ bands represent degradation products generated by autocatalysis or by other proteinases. Total AKT is shown as a loading control. **B and D**. Densitometric analysis. *, *p* ≤ 0.05; + TIMP-2 *vs*. the corresponding – TIMP-2 sample. These experiments were repeated twice with comparable results.

TIMP-2 activation of the Ras–ERK1/2 pathway is mediated by MT1-MMP through a proteolysis-independent mechanism [37]. To investigate whether the effect of TIMP-2 on AKT activation is also independent of the proteolytic activity of MT1-MMP, we tested the effect of Ilomastat (GM6001) on AKT activation in MT1-MMP expressing cells. Ilomastat, a broad-spectrum MMP inhibitor, inhibits MT1-MMP activity as efficiently as TIMP-2. We reasoned that if the effect of TIMP-2 on activation of AKT results from inhibition of MT1-MMP activity, Ilomastat would also activate AKT in MT1-MMP expressing cells. The results (Fig 4A and 4B), showed that Ilomastat (50 μM) did not induce AKT activation, indicating that TIMP-2-induced activation of AKT does not require inhibition of MT1-MMP activity. Moreover, Ilomastat completely blocked AKT activation by TIMP-2, a result consistent with our previous finding that Ilomastat inhibits TIMP-2 activation of ERK1/2 by competing with TIMP-2 for binding to MT1-MMP [37].

To further investigate the involvement of MT1-MMP activity in AKT activation by TIMP-2 we transiently transfected MCF-7 cells with an MT1-MMP mutant devoid of proteolytic activity (E240A). Cells transfected with wt MT1-MMP or the empty vector were used as positive and negative controls, respectively. TIMP-2 induced AKT activation in cells transfected with MT1-MMP E240A as efficiently as in wt MT1-MMP transfected cells but not in the control, empty vector transfectants (Fig 4C and 4D). Thus, the results of these two experiments showed that AKT activation by TIMP-2 requires TIMP-2 binding to MT1-MMP and is independent of the proteolytic activity of MT1-MMP.

### ERK1/2 but Not AKT Activation by TIMP-2/MT1-MMP Is Dependent on FGF Receptor

**Fig 5:**
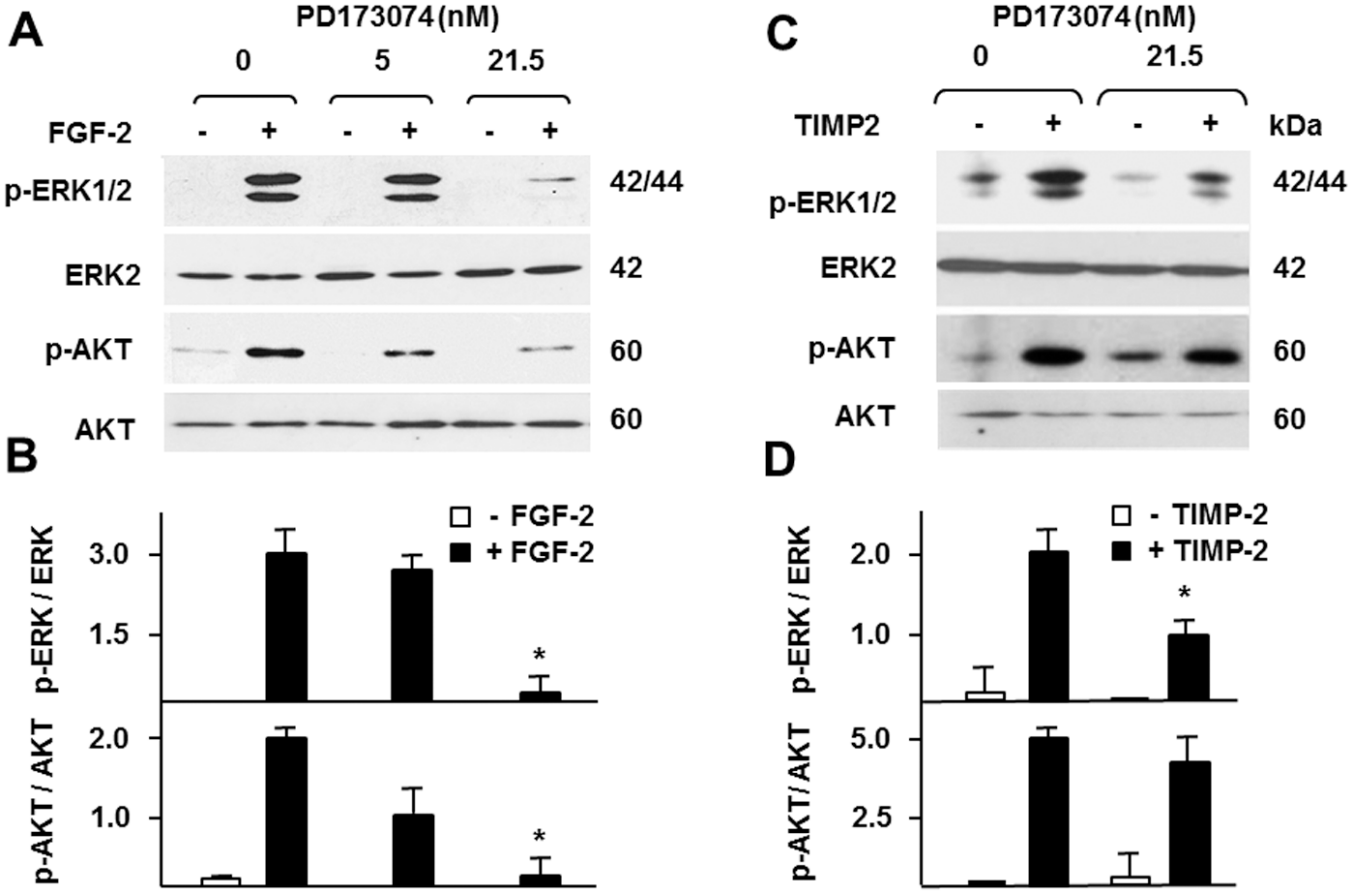
ERK1/2 but not AKT activation by TIMP-2/MT1-MMP is dependent on FGF receptor. Western blotting analysis of AKT and ERK1/2 activation (p-AKT; p-ERK1/2). MT1-MMP-expressing Tet-Off MCF-7 cells pretreated with the indicated concentrations of PD173074 or control vehicle (0) for 15 min were incubated with (**A**) recombinant FGF-2 (10 ng/ml; Akron Biotech, Boca Raton, FL, USA) or (C) TIMP-2 (100 ng/ml), or with control medium (-), for additional 15 min. **B** and **D**. Densitometric analysis of the blots shown in A and B, respectively. Total AKT and ERK2 are shown as loading controls. *, *p* ≤ 0.05, *vs*. the corresponding control with no PD173074. This experiment was repeated twice with comparable results.

MT1-MMP interacts with several transmembrane proteins that activate intracellular signaling [9,46–48], and TIMP-2 induces rapid tyrosine phosphorylation of cellular proteins with M_r_s ranging 100,000–200,000 [49], similar to those of receptor tyrosine kinases. Therefore, we hypothesized that TIMP-2 / MT1-MMP activation of ERK1/2 and AKT is mediated by a growth factor receptor. Because FGF receptors (FGFR) activate both of these signaling pathways, and our MT1-MMP transfectants express FGFR-1, -2 and -4 [50], we investigated the hypothesis that the effect of TIMP-2 on intracellular signaling is mediated by FGFR. For this purpose we used PD173074, a synthetic inhibitor of FGFR-1 [51]. Pretreatment of MT1-MMP expressing MCF-7 cells with PD173074 blocked FGF-2 activation of both AKT and ERK1/2 with a dose-dependent effect (Fig 5A and 5B). Similarly, PD173074 strongly inhibited TIMP-2 induced activation of ERK1/2; however, it had no such effect on AKT activation by TIMP-2 (Fig 5C and 5D), indicating that TIMP-2 interaction with MT1-MMP activates the ERK1/2 and AKT pathways by FGFR1-dependent and -independent mechanisms, respectively.

### TIMP-2 Activation of AKT Is Mediated by Ras

**Fig 6:**
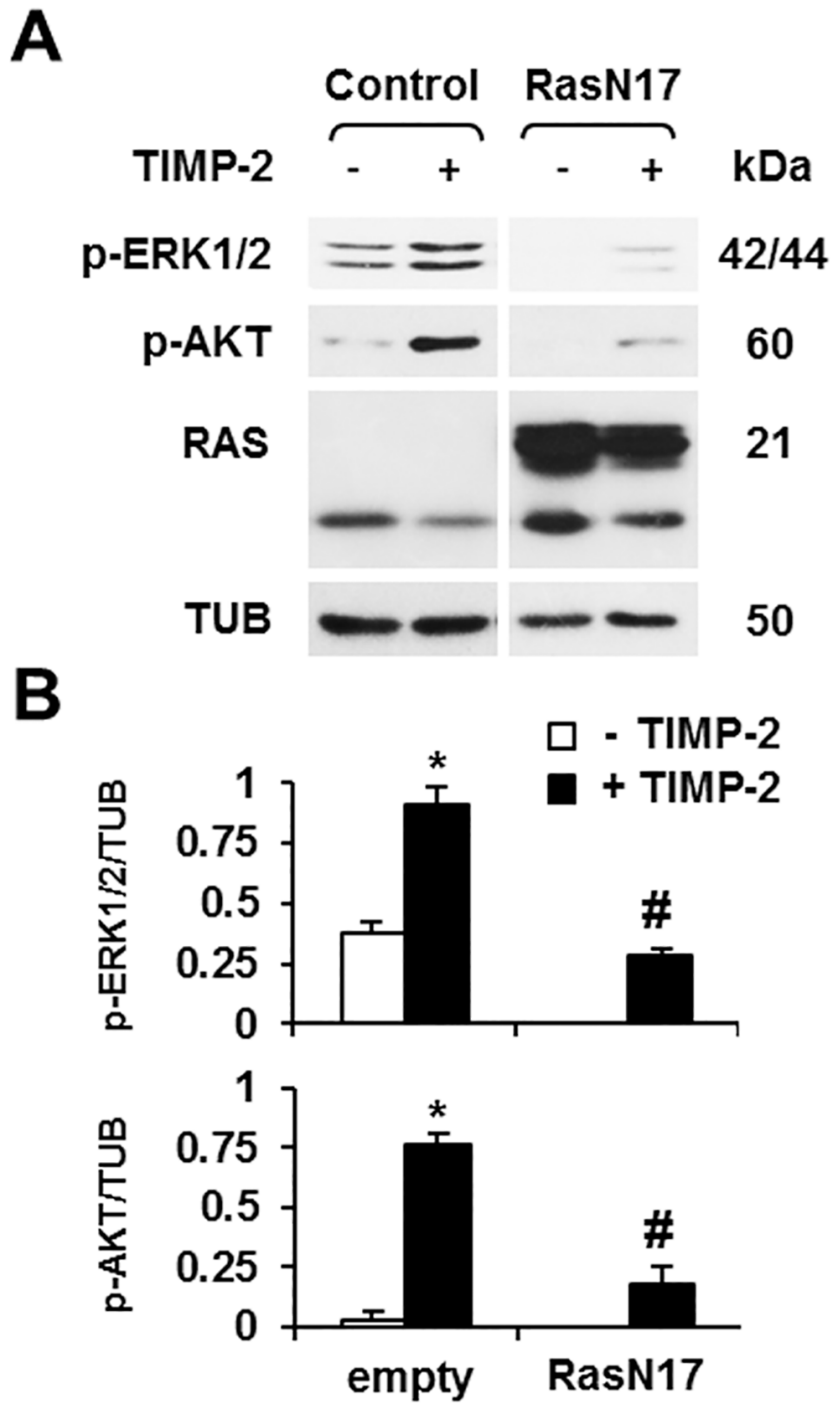
TIMP-2 activation of both ERK 1/2 and AKT is mediated by Ras. **A**. MT1-MMP-expressing Tet-Off MCF-7 cells transiently transfected with RasN17 cDNA or control, empty vector were incubated with TIMP-2 (100 ng/ml) for 15 min. Cell extracts were analyzed by Western blotting for ERK1/2 and AKT activation (p-ERK1/2; p-AKT), and for RasN17 expression using β-tubulin (TUB) as a loading control. B. Densitometric analysis. *, *p* ≤ 0.05, + TIMP-2 *vs*. the corresponding – TIMP-2 sample; # RasN17 *vs*. the corresponding empty vector control. This experiment was repeated three times with comparable results.

Our previous studies have shown that TIMP-2 binding to MT1-MMP induces Ras activation [37]. Because AKT can also be activated by Ras [52,53], we hypothesized that TIMP-2 activation of Ras results in the downstream activation of both ERK1/2 and AKT. To investigate this hypothesis we transiently transfected MT1-MMP expressing MCF-7 cells with RasN17, a dominant negative mutant of Ras that blocks activation of endogenous Ras [53]. Overexpression of RasN17 inhibited TIMP-2 induction of both AKT and ERK1/2, showing that AKT activation by TIMP-2 is mediated by Ras (Fig 6).

### TIMP-2 – MT1-MMP Interaction Induces Prosurvival Signaling through Both the ERK1/2 and AKT Pathways

**Fig 7:**
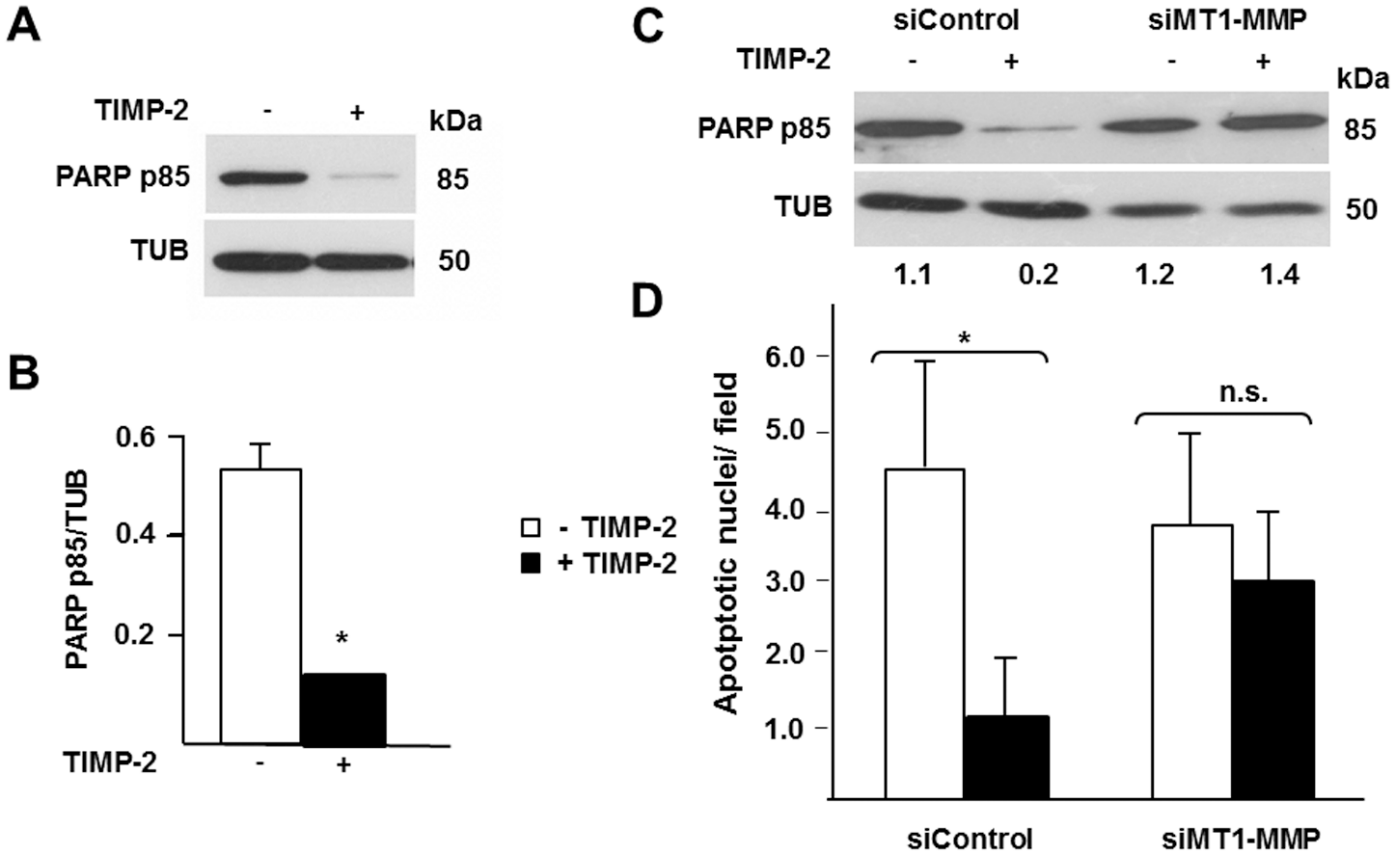
TIMP-2 – MT1-MMP interaction induces prosurvival signaling. Western blotting analysis of PARP degradation (PARP p85) in (**A**) MT1-MMP-expressing Tet-Off MCF-7 cells and (**C**) the MDA-MB-435 cells transiently transfected with MT1-MMP siRNA or control scrambled siRNA, shown in Fig 2. To induce apoptosis the cells were grown in serum-free medium in the presence (+) or absence (-) of TIMP-2 (100 ng/ml) as described in Materials and Methods. Beta tubulin (TUB) is shown as a loading control. **B**. Densitometric analysis of the blot shown in panel A. **C**. The numbers shown below the lower blot represent densitometric values of the PARP p85 bands normalized to the corresponding β-tubulin bands. **D**. Number of apoptotic nuclei / 10 X field in MDA-MB-435 cells transiently transfected with MT1-MMP siRNA or control, scrambled siRNA, and grown in serum-free medium in the presence (+) or absence (-) of TIMP-2 for 3 days as described in Materials and Methods. Apoptotic nuclei were characterized as described in Materials and Methods. *, *p* ≤ 0.05, + TIMP-2 *vs*. the corresponding – TIMP-2 samples; n.s., not significant. The experiment shown in A and B was repeated at least three times and the experiment shown in C and D twice, with comparable results.

To investigate the biological role of TIMP-2 – MT1-MMP activation of AKT we characterized its effect on apoptosis. AKT mediates prosurvival signaling in a variety of cell types, and is of particular importance in tumor cells [54]. Therefore, we hypothesized that TIMP-2 interaction with MT1-MMP generates prosurvival signaling. To investigate this hypothesis we serum starved MT1-MMP-expressing MCF-7 cells in the presence or absence of TIMP-2, and characterized apoptosis by Western blotting analysis of PARP degradation, a marker of apoptosis. As shown in Fig 7A and 7B), TIMP-2 dramatically reduced PARP degradation, indicating that TIMP-2 interaction with MT1-MMP generates prosurvival signaling.

To confirm that the effect of TIMP-2 on apoptosis is not a unique feature of our MCF-7 cell transfectants we used the MDA-MB-435 cells transfected with MT1-MMP siRNA described above. As shown in Fig 7C, TIMP-2 strongly inhibited PARP degradation in cells transfected with control siRNA, which constitutively express MT1-MMP, but had no such effect on MT1-MMP siRNA-transfected cells, which express very low levels of MT1-MMP (Fig 2).

In addition to PARP degradation, we also characterized the effect of TIMP-2 on the chromatin condensation and nuclear fragmentation typical of apoptosis. For this purpose we transfected MDA-MB-435 cells with MT1-MMP siRNA or control siRNA, serum-starved them in the presence or absence of TIMP-2, and measured apoptotic nuclei by DAPI staining as described in Materials and Methods. The results (Fig 7D) were consistent with those obtained by the analysis of PARP degradation. TIMP-2 dramatically decreased the number of apoptotic nuclei in cells transfected with control siRNA but had no such effect on MT1-MMP siRNA-transfected cells, which express virtually no MT1-MMP (Fig 2). Therefore, these results showed that TIMP-2 interaction with MT1-MMP protects tumor cells from apoptosis.

**Fig 8:**
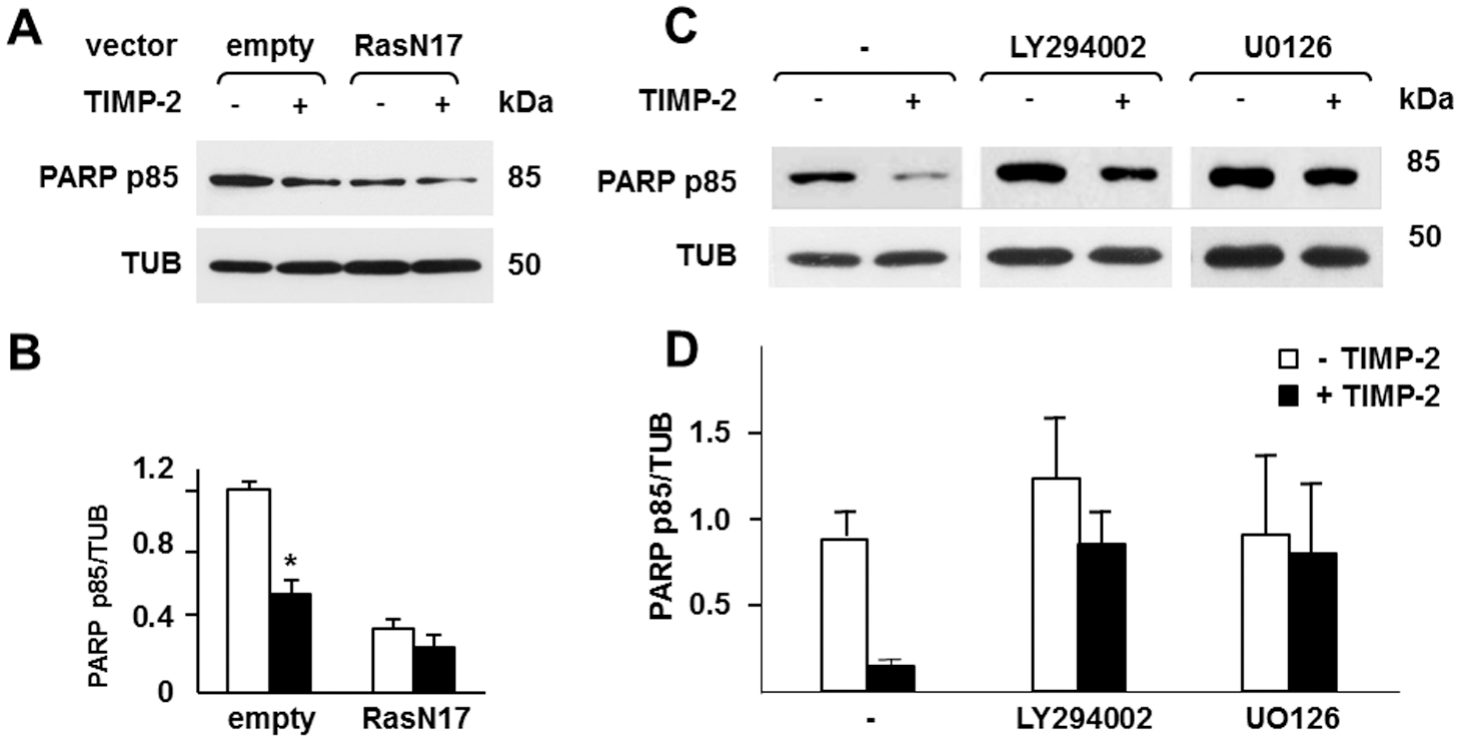
TIMP-2 – MT1-MMP interaction induces prosurvival signaling through Ras activation and both the ERK1/2 and AKT pathways. **A**. Western blotting analysis of PARP degradation (PARP p85) in MT1-MMP-expressing Tet-Off MCF-7 cells transiently transfected with RasN17 cDNA or control empty vector, grown in serum-free medium in the presence (+) or absence (-) of TIMP-2 (100 ng/ml) for 7 days. **B**. Densitometric analysis. **C**. Western blotting analysis of PARP degradation in MT1-MMP-expressing Tet-Off MCF-7 cells grown for 7 days in serum-free medium with LY294002 (10 μM) or U0126 (10 μM) or with control vehicle (-) in the presence (+) or absence (-) of TIMP-2 (100 ng/ml). **D**. Densitometric analysis. Beta tubulin (TUB) is shown as a loading control. *, *p* ≤ 0.05, + TIMP-2 *vs*. the corresponding – TIMP-2 sample. This experiment was repeated twice with comparable results.

To investigate the prosurvival signaling activated by TIMP-2 – MT1-MMP interaction we characterized the effect of inhibition of the ERK1/2 and AKT signaling pathways on apoptosis. We reasoned that blocking Ras activation, the common upstream activator of the two pathways, as well as the selective inhibition of either the downstream ERK1/2 or AKT pathway should abrogate the anti-apoptotic effect of TIMP-2. In a first set of experiments, cells transiently transfected with RasN17 or control empty vector were serum starved in the presence or absence of TIMP-2. As expected, TIMP-2 strongly reduced apoptosis in the control transfectants (Fig 8A and 8B). However, although after serum starvation apoptosis was downregulated in RasN17-transfected cells, treatment with TIMP-2 did not further reduce apoptosis, indicating that TIMP-2-activated survival signaling requires Ras activation. In a second set of experiments (Fig 8C and 8D), we serum starved the cells in the presence or absence of LY294002, a synthetic PI3K inhibitor that blocks AKT activation, and UO126, a MEK inhibitor that blocks ERK1/2 activation. As expected, both inhibitors upregulated apoptosis, but this effect was non-significantly reversed by addition of TIMP-2, indicating that the prosurvival signaling activated by TIMP-2 / MT1-MMP interaction is mediated by both the ERK1/2 and AKT pathways.

### TIMP-2 Interaction with MT1-MMP Activates Pro- or Anti-Apoptotic Signaling Depending on Context

**Fig 9:**
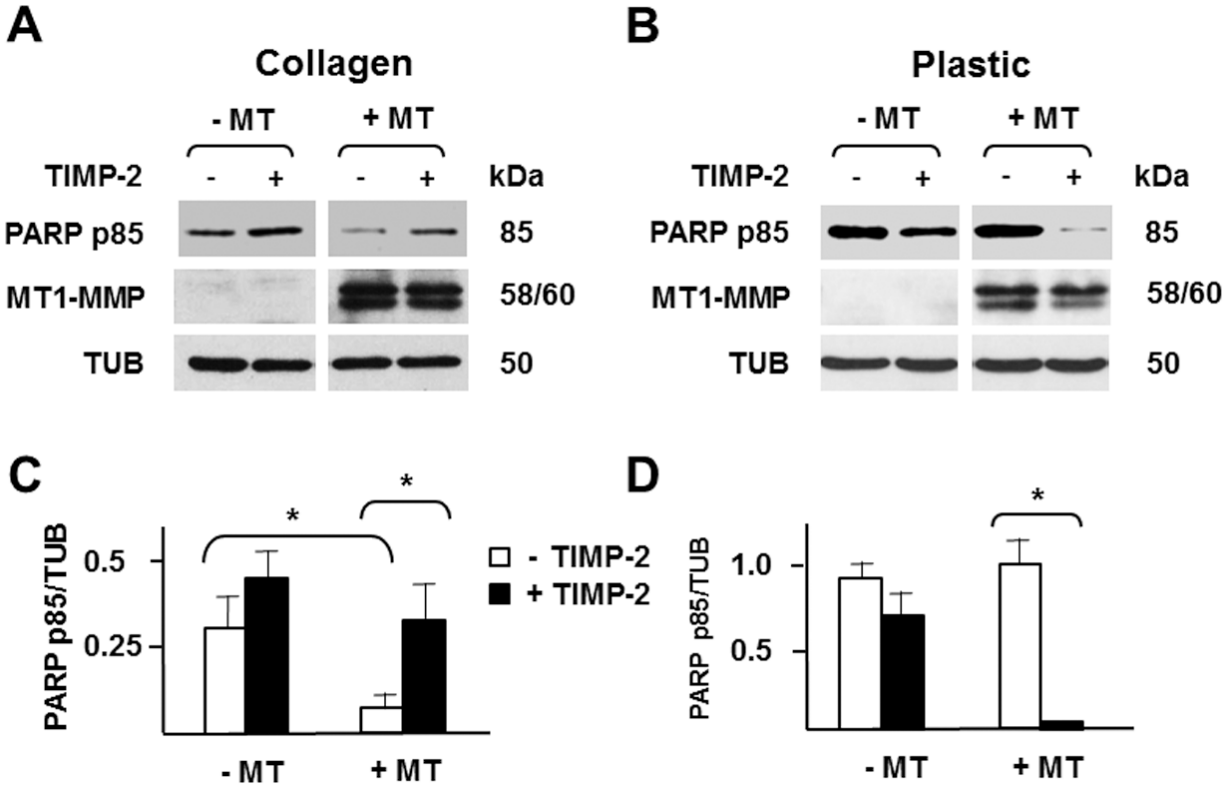
TIMP-2 interaction with MT1-MMP activates pro- or anti-apoptotic signaling depending on context. Western blotting analysis of PARP degradation (PARP p85) and MT1-MMP expression in MT1-MMP Tet-Off MCF-7 cells grown for 7 days in (**A**) 3D collagen gel or (**B**) on plastic, in the presence (-MT) or absence (+MT) of DOX with (+) or without (-) TIMP-2 (100 ng/ml). Beta tubulin (TUB) is shown as a loading control. **C and D**. Densitometric analysis. *, *p* ≤ 0.05. This experiment was repeated twice with comparable results.

Our observation that TIMP-2 induces prosurvival signaling contrasts with a previous finding that the proteolytic activity of MT1-MMP protects MCF-7 cells from collagen I-induced apoptosis in three-dimensional (3D) culture [55]. MT1-MMP degradation of collagen I abrogates apoptotic signaling generated by collagen I in epithelial cells. Because TIMP-2 inhibits MT1-MMP activity, it is expected to abrogate its prosurvival effect. However, our findings of the prosurvival signaling of TIMP-2 in 2D culture raised the question whether in3D culture the anti-apoptotic or anti-survival effect of TIMP-2 would be prevalent. Therefore, in parallel experiments we induced apoptosis either by growing cells in 3D collagen gel or by serum starvation. Cells expressing or devoid of MT1-MMP were grown in the presence or absence of TIMP-2 either as monolayers in plastic culture dishes in serum-free medium or in suspension in 3D collagen gel in complete medium. Consistent with previous findings [55], growth in 3D collagen induced apoptosis (Fig 9A and 9C) in cells devoid of MT1-MMP, and this effect was strongly reduced by MT1-MMP expression. Addition of TIMP-2 to the culture medium neutralized the anti-apoptotic effect of MT1-MMP, and increased apoptosis to a level comparable to that of cells devoid of MT1-MMP. In contrast, TIMP-2 dramatically reduced apoptosis, and virtually suppressed it in serum-starved MT1-MMP expressing cells grown on plastic (Fig 9B and 9D), but had no such effect on cells devoid of MT1-MMP. Therefore, these results showed that TIMP-2 and MT1-MMP can have both pro- and anti-apoptotic signaling depending on the environment and apoptotic stimulus.

## Discussion

We have previously shown that TIMP-2 binding to MT1-MMP activates the Ras-ERK1/2 pathway through a proteolysis-independent mechanism. Here we show that TIMP-2 interaction with MT1-MMP activates AKT by a similar mechanism and provides tumor cells with prosurvival signaling.

The data presented show several aspects of the mechanism of AKT activation by TIMP-2. Like ERK1/2, AKT is activated by TIMP-2 concentrations in the range of those found in tissues or biological fluids (10–100 ng/ml, or 0.4–4.0 nM) [40–43] and similar to the K_D_ of TIMP-2 for MT1-MMP (0.77–2.54 nM) [37,44,56]. TIMP-2-induced activation of AKT is not mediated by inhibition of MT1-MMP proteolytic activity but requires TIMP-2 binding to MT1-MMP. Two findings support this conclusion. Ilomastat, a broad-spectrum MMP, does not induce AKT activation although it inhibits MT1-MMP activity as efficiently as TIMP-2, showing that MT1-MMP activation of AKT is not mediated by inhibition of MT1-MMP activity. Moreover, Ilomastat prevents AKT activation by TIMP-2. This observation is consistent with the well-established concept that Ilomastat, as well as other hydroxamic acid-based MMP inhibitors, competes with TIMP-2 for binding to MT1-MMP, and thus prevents TIMP-2 from binding to MT1-MMP [45]. Our previous studies have shown that Ilomastat dose-dependently inhibits TIMP-2 binding to MT1-MMP and blocks TIMP-2 induced activation of ERK1/2 [37]. In addition, TIMP-2 activation of AKT is mediated by proteolytically inactive MT1-MMP (MT1-MMP E240A). We have previously shown that TIMP-2 binds to MT1-MMP E240A mutant as well as to wt MT1-MMP, and that mutant TIMP-2 devoid of MMP inhibitory activity (Ala-TIMP-2) binds to MT1-MMP and activates ERK1/2 as efficiently as wt TIMP-2 [37]. Thus, AKT activation is mediated by TIMP-2 interaction with the MT1-MMP catalytic domain and independent of the proteolytic activity of MT1-MMP.

Our previous studies have shown that the TIMP-2 concentration that activates Ras-ERK1/2 signaling also inhibits MMP-2 activation by the MT1-MMP Tet-Off transfectants we used in the present manuscript [37]. However, these cells do not express MMP-2, and TIMP-2 induction of Ras-ERK1/2 activation occurs in the absence of MMP-2 [37,38], indicating that MMP-2 is not involved in MT1-MMP control of AKT signaling.

In addition to similarities between ERK1/2 and AKT activation by TIMP-2 / MT1-MMP, we also found differences. Although both ERK1/2 and AKT activation are mediated by Ras, TIMP-2 activation of ERK1/2 is inhibited by PD173074, a specific inhibitor of FGFR1, whereas AKT activation is not inhibited. The immediate upstream activator of AKT is phosphoinositide 3-kinase (PI3K), a heterodimeric enzyme consisting of a p85 regulatory subunit that harbors an SH2 domain, and a catalytic p110 subunit. Unlike ERK1/2, PI3K can be activated through different independent mechanisms, either triggered by recruitment of the p85 subunit to a tyrosine kinase receptor and consequent activation of the catalytic p110 subunit or, alternatively, p110 activation can be mediated by Ras independently of p85 [52,53,57]. It is not clear whether the mechanism of election is determined by specific conditions or linked to a specific biological response. We speculate that AKT activation by TIMP-2 / MT1-MMP is mediated by a different, proteolysis-independent mechanism that may result in Ras activation at a location on the plasma membrane different from that of FGFR. This hypothesis implies that MT1-MMP can activate intracellular signaling through multiple mechanisms, and is supported by the well-established notion that MT1-MMP interacts with a variety of cell surface and transmembrane proteins that can mediate activation of intracellular signaling [9,46]. Thus, it is possible that AKT activation is mediated by TIMP-2 / MT1-MMP interaction with a transmembrane protein (e.g. growth factor receptor, integrin) other than FGFR1. This hypothesis warrants further investigation.

TIMP-2 binding to MT1-MMP controls several important cell functions by a proteolysis-independent mechanism. Our previous work has shown that MT1-MMP – TIMP-2 interaction upregulates cell proliferation and migration [37]. Our present data show TIMP-2 binding to MT1-MMP induces anti-apoptotic signaling through the proteolysis-independent activation of Ras and both the downstream ERK1/2 and AKT pathways. We also found that inhibition of Ras activation protects the cells from apoptosis induced by serum starvation, indicating that Ras can relay both pro- and anti-apoptotic signaling. This observation may seem inconsistent with the well-established notion that constitutive activation of Ras promotes oncogenesis by stimulating cell proliferation and survival. However, a number of independent studies have shown that ectopic expression or endogenous activation of Ras is a critical step for the initiation of a death program by tumor cells in response to pharmacological or environmental insults such as growth factor (serum) deprivation. The mechanism(s) that determine the switch between the pro- and anti-apoptotic effect of Ras activation remain unclear, and may depend on the balance between Ras isoforms and interaction between different signaling pathways (reviewed in [52]).

Like Ras, TIMP-2 can also have both pro- and anti-apoptotic effects, depending on the cellular microenvironment and apoptotic stimulus. Consistent with previous reports [55], we found that MT1-MMP protects tumor epithelial cells from collagen I induced apoptosis. In the presence of collagen I TIMP-2 upregulates apoptosis as it blocks the protective effect of MT1-MMP. However, in the absence of collagen I (cells grown on plastic) TIMP-2 protects the cells from starvation-induced apoptosis by activating prosurvival signaling through MT1-MMP. Although both experimental conditions are artificial, the growth of cells in 3D collagen gels mimics the setting of invasive carcinoma cells in a stroma, where they become exposed to a pro-apoptotic extracellular matrix. Conversely, cells grown on plastic secrete and adhere to the extracellular matrix they produce, mimicking the condition of epithelial cells adherent to their own basement membrane. In this setting TIMP-2 interaction with MT1-MMP protects the cells from apoptosis by activating prosurvival pathways. This finding can explain the paradoxal observation that, while high levels of MT1-MMP are associated with aggressiveness in a variety of human malignancies [26],[27], high levels of TIMP-2 also correlate with a poor prognosis. Indeed, in some tumors a negative outcome correlates more closely with TIMP-2 than with MT1-MMP levels [28–35], and high TIMP-2 levels in primary breast carcinomas are associated with the development of distant metastases [30,36].

In conclusion, the data reported show a novel biological function of MT1-MMP and TIMP-2 that can have an important role in tumor biology. The pharmacological inhibition of TIMP-2 – MT1-MMP activation of pro-survival signaling can provide a novel approach to the treatment of tumor development and progression.
